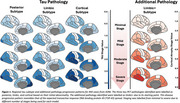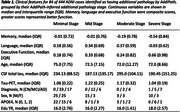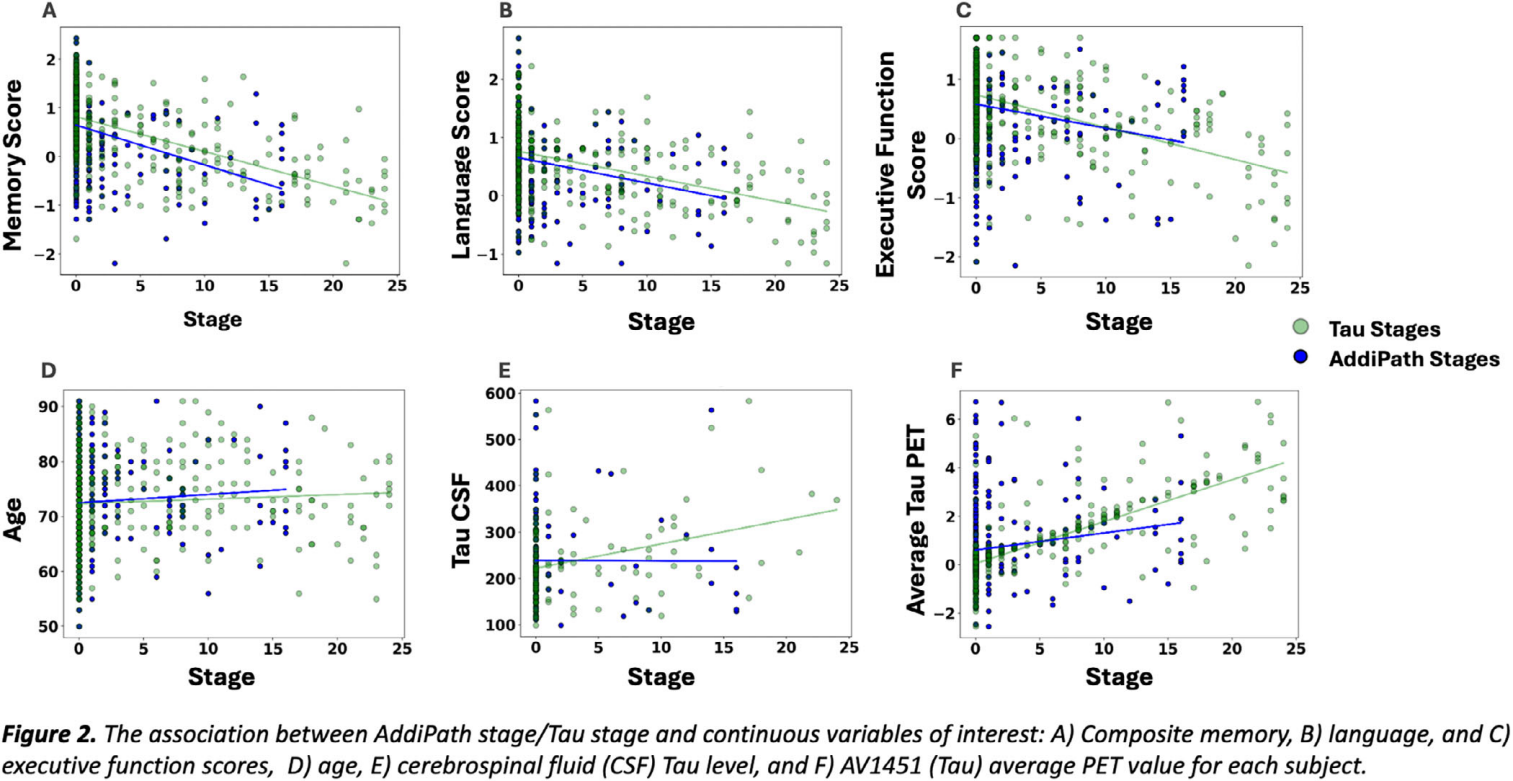# Uncovering regional atrophy not explained by tau deposition using Additional Pathology Inference (AddiPath)

**DOI:** 10.1002/alz70862_110289

**Published:** 2025-12-23

**Authors:** Lawrence P Binding, Mihaela Croitor, Christopher S Parker, Isaac Llorente Saguer, Neil P Oxtoby, Daniel C Alexander, Alexandra L Young

**Affiliations:** ^1^ UCL Hawkes Institute and Department of Computer Science, University College London, London UK

## Abstract

**Background:**

Mixed pathology is prevalent in Alzheimer’s disease (AD) but difficult to detect *in vivo* as biomarkers for several additional pathologies are in their infancy. Cortical thickness (CT) atrophy reflects both tau deposition and additional pathologies (e.g., TDP‐43). Specific biomarkers like tau‐PET may help disentangle these contributions. We introduce Additional Pathology Inference (AddiPath), software designed to identify disease biomarker changes not explained by primary pathology, and apply it to identify CT changes unexplained by tau deposition on tau‐PET.

**Method:**

Paired tau‐PET and CT data from 444 ADNI subjects across 8 bilateral meta‐regions were analysed. Subtype and Stage Inference (SuStaIn) applied to tau‐PET data identified tau pathology subtypes with distinct progression patterns. AddiPath was then applied to learn the relative contributions of tau and additional pathology to regional CT values. AddiPath simultaneously estimated a scaling parameter determining the contribution of the regional SuStaIn‐predicted tau‐PET value to changes in CT, and the regional progression pattern of the additional CT changes that were not explained by tau‐PET. Regression analysis was utilised to analyse the effects of AddiPath stages on clinical variables of interest.

**Result:**

SuStaIn revealed posterior, limbic, and cortical subtypes of tau deposition (Figure 1), AddiPath uncovered an additional pathology progression pattern (Figure 1) beginning in the entorhinal cortex, spreading to the medial temporal lobe, and then across the cortex. 84 individuals (50 mild cognitive impairment; 33 dementia; 1 control) were labelled by AddiPath as having additional pathology (Table 1). Increased AddiPath stages were associated with worse composite memory (*p*<0.001), language (*p* = 0.001) and executive function (*p*<0.001) scores. Greater tau‐PET pathology stages correlated with CSF total tau (*p* <0.001) and tau‐PET (*p* <0.001), but AddiPath stages did not (*p* = 0.860, *p* = 0.475), confirming that AddiPath‐inferred additional pathology was unrelated to tau load (Figure 2).

**Conclusion:**

We introduced AddiPath, a method for disentangling the contributions of additional pathology from primary pathology. AddiPath uncovered changes in CT not explained by tau deposition in the limbic lobe, possibly indicating TDP‐43 deposition. This approach could improve patient selection for clinical trials and treatment stratification. Future work will replicate this finding across additional cohorts and test the applicability of AddiPath to other biomarkers.